# Mapping the epigenomic landscape of human monocytes following innate immune activation reveals context-specific mechanisms driving endotoxin tolerance

**DOI:** 10.1186/s12864-023-09663-0

**Published:** 2023-10-07

**Authors:** Harindra E. Amarasinghe, Ping Zhang, Justin P. Whalley, Alice Allcock, Gabriele Migliorini, Andrew C. Brown, Giuseppe Scozzafava, Julian C. Knight

**Affiliations:** 1 Wellcome Centre for Human Genetics, Nuffield Department of Clinical Medicine, University of Oxford, Oxford, OX3 7BN UK; 2 Chinese Academy of Medical Science Oxford Institute, University of Oxford, Oxford, OX3 7BN UK

**Keywords:** Endotoxin tolerance, Human monocytes, Context-specificity, Chromatin accessibility, Enhancer RNA, Expression quantitative trait loci

## Abstract

**Background:**

Monocytes are key mediators of innate immunity to infection, undergoing profound and dynamic changes in epigenetic state and immune function which are broadly protective but may be dysregulated in disease. Here, we aimed to advance understanding of epigenetic regulation following innate immune activation, acutely and in endotoxin tolerant states.

**Methods:**

We exposed human primary monocytes from healthy donors (*n* = 6) to interferon-γ or differing combinations of endotoxin (lipopolysaccharide), including acute response (2 h) and two models of endotoxin tolerance: repeated stimulations (6 + 6 h) and prolonged exposure to endotoxin (24 h). Another subset of monocytes was left untreated (naïve). We identified context-specific regulatory elements based on epigenetic signatures for chromatin accessibility (ATAC-seq) and regulatory non-coding RNAs from total RNA sequencing.

**Results:**

We present an atlas of differential gene expression for endotoxin and interferon response, identifying widespread context specific changes. Across assayed states, only 24–29% of genes showing differential exon usage are also differential at the gene level. Overall, 19.9% (6,884 of 34,616) of repeatedly observed ATAC peaks were differential in at least one condition, the majority upregulated on stimulation and located in distal regions (64.1% vs 45.9% of non-differential peaks) within which sequences were less conserved than non-differential peaks. We identified enhancer-derived RNA signatures specific to different monocyte states that correlated with chromatin accessibility changes. The endotoxin tolerance models showed distinct chromatin accessibility and transcriptomic signatures, with integrated analysis identifying genes and pathways involved in the inflammatory response, detoxification, metabolism and wound healing. We leveraged eQTL mapping for the same monocyte activation states to link potential enhancers with specific genes, identifying 1,946 unique differential ATAC peaks with 1,340 expression associated genes. We further use this to inform understanding of reported GWAS, for example involving *FCHO1* and coronary artery disease.

**Conclusion:**

This study reports context-specific regulatory elements based on transcriptomic profiling and epigenetic signatures for enhancer-derived RNAs and chromatin accessibility in immune tolerant monocyte states, and demonstrates the informativeness of linking such elements and eQTL to inform future mechanistic studies aimed at defining therapeutic targets of immunosuppression and diseases.

**Supplementary Information:**

The online version contains supplementary material available at 10.1186/s12864-023-09663-0.

## Introduction

Monocytes show a remarkable degree of cellular heterogeneity and plasticity which allows them to play vital and diverse roles in the innate immune system, recognising and responding to infection [1, 2]. Recognition of pathogen-associated molecular patterns such as bacterial endotoxin (lipopolysaccharide, LPS), viral double-stranded RNA, fungal β-glucan, or molecules released by damaged cells, triggers a rapid and potent inflammatory response [3, 4] through activation of diverse signalling pathways involving nuclear factor-kappa B (NF-κB), Janus kinase (*JAK*)-signal transducer and activator of transcription (*STAT*), mitogen-activated protein kinases (MAPKs) and IFN regulatory factor (IRFs). These regulate inflammatory mediators (cytokines, chemokines, cell adhesion molecules and immunoreceptors), phagocytosis, cell locomotion, antigen presentation as well as immune tolerance [5, 6].

Monocytes can develop an immunological memory subsequent to stimulation with pathogen moieties or vaccinations, a process known as trained immunity. This is a long-term functional adaptation which allows the cells to respond to a secondary stimulation at an enhanced level [7–9]. Conversely, monocytes can respond with reduced levels of pro-inflammatory cytokine production to repeated pathogen exposure (experimentally, through stimulation with endotoxins and other TLR ligands), a response referred to as endotoxin tolerance. Here cells undergo extensive epigenetic, transcriptional and metabolic reprogramming to be less responsive or desensitise to any subsequent endotoxin stimulus, a regulatory mechanism of counteracting excessive inflammation [10–13]. Epigenetic regulation is also important for interferons (IFNs), pleiotropic cytokines that play an important coordinating role in the innate antiviral response, limiting viral replication [14–16].

Immune homeostasis in monocytes, the balance between trained immunity and tolerance, has been identified as dysregulated in many disease states, including following infection in COVID-19 and sepsis, and non-infectious diseases such as trauma, acute coronary syndrome, cancer, diabetes and pancreatitis [17–20]. In sepsis for instance, some individuals develop a predominant immunosuppressive state which can be maladaptive and contribute to increased mortality, and shows enrichment of an endotoxin tolerance gene signature [21, 22].

Apart from the type of pathogen or endotoxin, other factors such as cell or tissue type [23, 24], host heterogeneity such as genetic predisposition, epigenetics, ethnicity, age, and gender, also contribute to observed variability in immune response states [25, 26]. At the molecular level, these differences may manifest for example as expression quantitative trait loci (eQTL) that identify genetic associations with differences in gene expression between individuals, and are highly context-specific dependent on activation state and time [27–31]. Mapping and understanding such context-specific eQTL is important to interpret genome-wide association studies of disease as the vast majority of GWAS associated genetic variants are located in the non-coding genome [29, 32]. GWAS variants are significantly enriched for monocyte eQTL in a context-specific manner [28], providing evidence to identify the genes and pathways driving observed genetic associations with disease. Expression-associated SNPs are enriched in histone marks for active enhancers, and open chromatin regions [30, 33–35] raising the hypothesis that the specificity of such eQTL may depend on changes occurring in chromatin remodelling and accessibility, and that a combination of genetic and epigenetic processes modulate our individual immune and inflammatory response, allowing for example monocytes to function with varying degrees of plasticity and specificity. Identifying context-specific regulatory elements based on epigenetic signatures for chromatin accessibility and modifications, together with understanding of the extent and nature of regulatory non-coding RNAs (ncRNA), provides an opportunity to understand possible mechanisms underlying such eQTL. More broadly, such data can inform mechanisms of regulation of response to innate immune activation in monocytes and how this may be dysregulated in disease [12].

Here we sought to produce an atlas of the epigenomic response to endotoxin in primary human monocytes, acutely and for two models of endotoxin tolerance: repeated stimulation and prolonged exposure to endotoxin [13, 28, 36]. We complemented this with analysis of response to IFNγ. We aimed to first define the context-specific transcriptome and regulatory genomic landscape in these states based on analysis of differential gene expression, alternative splicing/differential exon usage, ncRNAs, chromatin accessibility and informative enhancer elements. We then sought to leverage such data to functionally interpret eQTL we had previously reported for these activation states as well as identify novel context-specific regulatory events that may impact on the functional consequences of such states and their dysregulation.

## Results

### Experimental design and cohort to investigate epigenetic and transcriptomic changes induced by innate immune activation

**Fig. 1:**
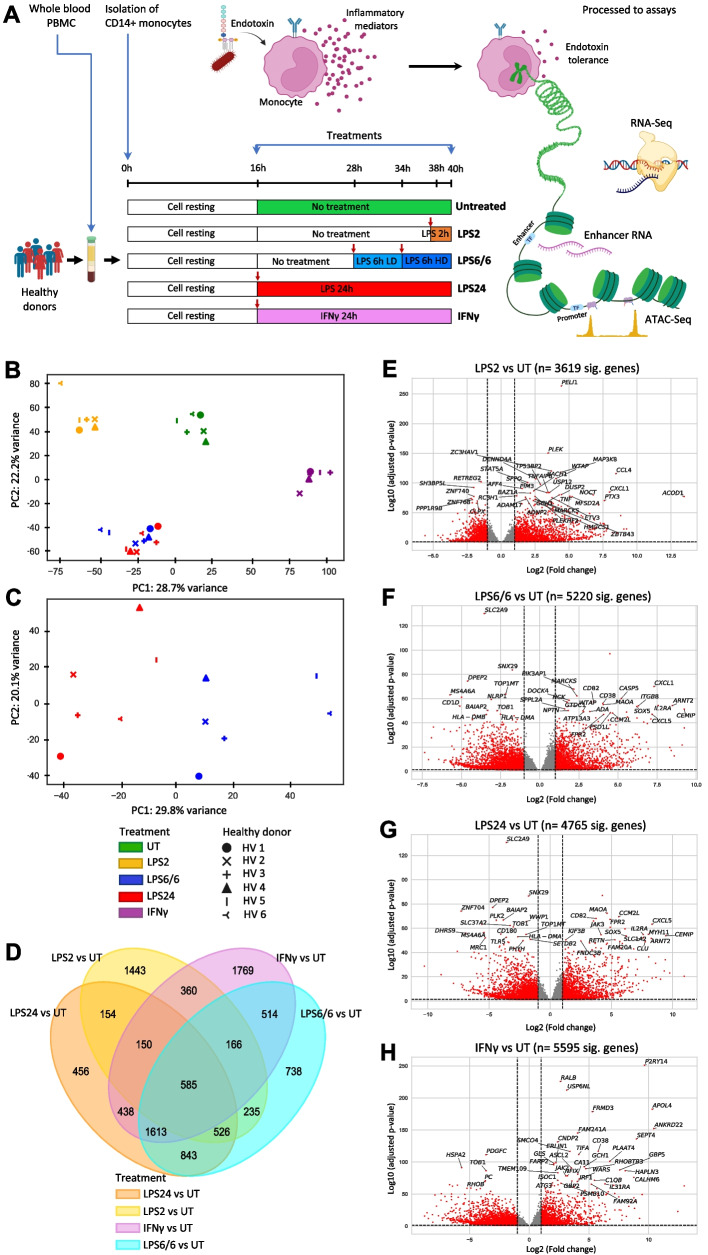
An overview of the experiment and context-specific transcriptomes of human primary monocytes. **A** Study design. Timeline of monocyte isolation, culture, stimulations and sampling for assays. CD14 + monocytes were extracted from whole blood PBMCs from six healthy donors and cultured for 16 h prior to immune stimulation with a single dose of LPS 20 ng/mL for 2 h (LPS 2) or for 24 h (LPS 24); low dose 2 ng/mL for 6 h then 20 ng/mL LPS (LPS6/6); or IFNγ 20 ng/mL for 24 h (IFNγ). Red arrows indicate the time point at which either LPS or IFNγ was added. All samples were harvested at the same time and processed for RNA-Seq and ATAC-Seq. Cell schematic illustrates the innate immune response of monocytes when encounter with a pathogen, development of tolerance and assays used to investigate epigenetic mechanisms. **B** Principal component analysis (PCA) of gene expression data across six healthy donors and five innate immune activation states. Each symbol represents an individual and colours indicate different states. **C** PCA showing variation in gene expression between LPS6/6 and LPS24, endotoxin tolerant states. **D** Venn diagram shows the numbers of differentially expressed genes overlap between activation states (UT, LPS2, LPS6/6, LPS24 and IFNγ). **E**–**H** Volcano plots illustrating top differentially expressed genes in red (fold change > 2 and adjusted p-values (FDR) < 0.05) in each treatment condition compared to the naïve untreated monocytes: (**E**) LPS2 vs UT, (**F**) LPS6/6 vs UT, (**G**) LPS24 vs UT and (**H**) IFNγ vs UT

We aimed to define the transcriptomic and regulatory genomic landscape of human primary monocytes under different conditions of innate immune activation and tolerance using CD14^+^ monocytes isolated from whole blood peripheral blood mononuclear cells (PBMCs) of six healthy donors of British Caucasian ancestry, with equal numbers of males and females. We exposed monocytes to differing combinations of LPS to investigate differential inflammatory responses: acute response by 2 h incubation with high dose LPS (20 ng/ml) (LPS2); and two models of tolerance namely 24 h incubation with high dose LPS (LPS24), or 6 h low dose LPS (2 ng/ml) followed by 6 h high dose of LPS (LPS6/6). To further model inflammation states that may occur following infection a sample of monocytes were incubated for 24 h with IFNγ, a potent inducer of anti-viral and anti-microbial responses in monocytes. To allow comparison with the naïve state of the same individuals, another sample of monocytes were left untreated (UT) (Fig. 1A). From these monocytes we performed assay for transposase-accessible chromatin sequencing (ATAC-seq) and total RNA sequencing (RNA-seq). Using this information, we profiled differential chromatin accessibility, differential transcript expression, enhancer RNA, non-coding and coding RNA to investigate how monocyte response changes from the naïve state with differing conditions of endotoxins and IFNγ.

### Context-specificity in differential gene expression to endotoxin

We first investigated global differences in gene expression between comparator groups using total RNA sequencing and found clear separation of naïve untreated monocytes from the stimulated (LPS or IFNγ) monocytes on principal components analysis (PCA) (Fig. 1B). Of the stimulated monocytes, LPS2, tolerance (LPS24 and LPS6/6) and IFNγ treatments show distinct differences and clear clustering of samples. Comparing with other treatment modalities, we observed minimal variation between LPS6/6 and LPS24 monocyte groups (Fig. 1B). However, restricting PCA to these treatment groups showed a clear separation on principal component (PC) 1, comprising ~ 30% of the variance (Fig. 1C).

We identified a total of 21,298 genes comprising of both differential (FDR < 0.05 and absolute |log2 fold change|> 1) and non-differential expression for each treatment condition. 17% (3,619 out of 21,298) of genes were differentially expressed in the acute LPS response group (LPS2) compared to the naïve untreated group (Fig. 1D and E; Table S1). Of these differentially expressed genes, 39.9% (1,443 out of 3,619) genes were unique to LPS2 condition (Fig. 1D). We also identified 24.5% (5,220 out of 21,298), 22.4% (4,765 out of 21,298), and 26.3% (5,595 out of 21,298) genes to be differentially expressed in LPS6/6, LPS24, and IFNγ conditions, respectively (Fig. 1F-H; Table S1) of which 14.1% (738 out of 5,220), 9.6% (456 out of 4,765), and 31.6% (1,769 out of 5,595) of genes were differentially expressed only in LPS6/6, LPS24, and IFNγ conditions, respectively (Fig. 1D).

To investigate similarities and differences in acute response to that seen with prolonged or repeated exposure to endotoxin, we compared LPS24 with LPS2 and identified 5,600 genes (Fig. S1A; Table S1) of which 20.4% (1,141 out of 5,600) were only observed in that contrast (Fig. S1D). We found that 5,659 genes were differentially expressed in LPS6/6 vs LPS2 (Fig. S1B and D) and 22.2% (1,256 out of 5,659) only observed in that contrast (Fig. S1D). There was a significant overlap of genes between LPS6/6 vs LPS2 and LPS24 vs LPS2 contrasts (4,144 genes, 58.2%) (Fig. S1D) with 1,051 genes differentially expressed between LPS24 and LPS6/6 treatment conditions (Fig. S1C). Of this 1,051, 15.3% (*n* = 161) genes were unique to LPS24 and LPS6/6 contrast only (Fig. S1D).

TNF expression was reduced in both models of endotoxin tolerance. Relative to the cells upon acute LPS response (2 h LPS treatment), we observed similar transcriptomic changes in cells upon endotoxin tolerance (6 h LPS Low Dose + 6 h High Dose) and LPS 24 h treatment, which is in line with the markedly reduced TNF signalling. The dynamic expression changes of hallmark genes upon acute immune response and endotoxin tolerance are shown in Fig. S1E.

To compare the gene signatures between proinflammatory and anti-inflammatory macrophage lineages and LPS tolerance, we used the downregulated genes in M2 vs. M1 of monocyte-derived macrophages (FDR-adjusted *P* < 0.01, fold change > 2) [37], and determined that 287 out of 1,080 genes (26.6%) overlapped with DE genes upon endotoxin tolerance (Fig. S2A), 56.6% of which showed consistent expression changes in both M2 vs. M1 and LPS tolerance, including genes encoding pro-inflammatory cytokines such as *TNF-α, IL-1α* and *IL-6* (Fig. S2B). However, there was no correlation of DE genes identified from macrophage polarisation (M2 vs. M1) and LPS treatments (LPS6/6 vs. LPS2, or LPS24 vs. LPS2) (Fig. S2C left panels), indicating both shared and distinct transcriptomic regulations among these different macrophage subsets.

Alternative splicing is recognised to occur extensively as part of the response to endotoxin in monocytes and macrophages [38–40]. We confirmed this in our dataset with substantial differences in abundance of alternatively spliced isoforms on LPS induction. Differences were also seen in our models of tolerance. Overall, on LPS6/6 treatment 75% (3,432 out of 4,541) of genes that use at least one differential exon not differentially expressed at the gene level, and only 25% (1,109 out of 4,541) of genes showing both differential expression at the gene level as well as differential exon usage (Fig. S3A; Table S2). These include the BAF chromatin remodelling complex subunit gene *BCL7C* (Fig. S3B) while the transcription factor *MYCL* showed differences specific to acute endotoxin induction (Fig. S3C).

### Context-specific epigenetic changes with immune stimulation state

**Fig. 2:**
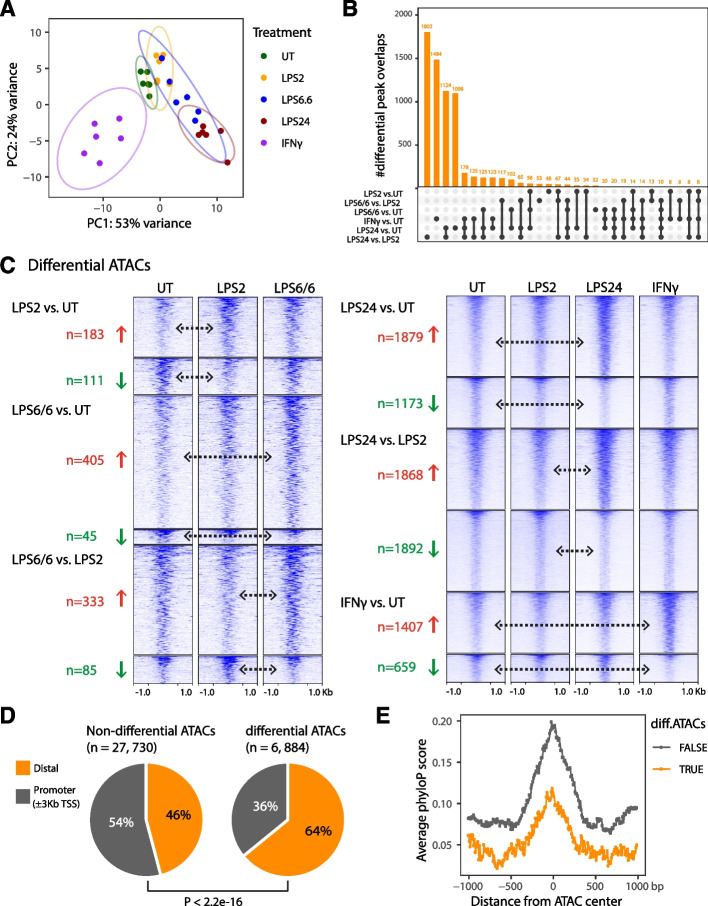
Immune stimulation conditions differentiate subsets of ATAC regions that are less conserved relative to the steady-state open chromatin. **A** PCA showing the chromatin accessibility in naïve or immune-stimulated cells. Each dot represents an independent sample and colours indicate different treatment conditions. **B** Upset plot of the number of differential ATAC peaks upon different treatment conditions. The filled dots in bottom section indicate peaks that are shared or unique to the corresponding contrasts listed on the left. The overlaps were determined and plotted using intervene (v0.6.4). **C** Heatmaps showing the normalized signals at differential ATAC peaks identified upon different treatment conditions. The directions of change (upregulation or downregulation) and number of differential ATAC peaks were highlighted in red and green. The ATAC mean signals across the donors were plotted with a ± 1 Kb window using deepTools. **D** Pie charts showing the fractions of ATAC peaks (left: non-differential ATACs; right: differential ATACs) in gene promoter regions (grey; ± 3 Kb of TSS) and distal regions (orange). **E** Average PhyloP conservation scores of the ± 1 Kb genomic regions centered on differential ATAC peaks (orange) and non-differential peaks (grey). The PhyloP scores for each region were calculated in 10-bp bins using bigWigAverageOverBed (see Methods)

We next investigated how endotoxin response varied at the epigenetic level by analysing differences in chromatin accessibility using ATAC-seq. PCA of overall variance across all samples revealed clustering by treatment condition (Fig. 2A). We found that the majority of variance was explained by PC1 (53% of variance), which separated IFNγ samples from the rest of the treatment types, with further stratification between naïve and LPS conditions, and within LPS conditions of stimulation. We found 19.9% (6,884 out of 34,616) of ATAC peaks called in ≥ 30% of all samples (denoted as recurrent) were differential in at least one of the treatment conditions, and the majority of these differential ATACs were identified upon LPS24 and/or IFNγ treatment (Fig. 2B).

Compared to naïve, untreated monocytes, we observed the highest number of differential chromatin accessibility changes after 24 h exposure to LPS (LPS24) with 3,052 differential ATAC peaks, including both upregulated (61.6%; 1,879 out of 3,052) and downregulated (38.4%; 1,173 out of 3,052). Similarly, when compared with the acute state (LPS2), we found that LPS24 showed the highest number of chromatin accessibility changes (*n* = 3,760), with equal numbers of upregulated (49.7%; 1,868 out of 3,760) and downregulated (50.3%; 1,892 out of 3,760) regions. The highest proportion of upregulated chromatin accessibility was seen for LPS6/6 vs UT condition with 90% upregulated differential ATAC peaks (405 out of 450) and 79.7% (333 out of 318) upregulated differential ATAC peaks for LPS6/6 vs LPS2. Similar to LPS, we found that the majority of differential ATAC peaks following IFNγ were also upregulated (68.1%, 1,407 out of 2,066; Fig. 2C).

### Immune stimulations differentiate subsets of ATAC regions that are less conserved relative to the steady-state open chromatin

We next classified the ATAC peaks into two groups based on their distances to the nearest transcription start site (TSS), and identified that the majority of differential ATACs (64.1%; 4,413 out of 6,884) resided in distal regions (> 3 Kb away from annotated TSSs), relative to 45.9% (12,730 out of 27,730) of the non-differential ATAC peaks (*P* < 2.2e-16, Fisher’s exact test); Fig. 2D). We further used the phyloP score as a measure of evolutionary conservation for the ATAC peaks (*Methods*). We determined the sequence conservation of the ± 1 kb region centred on the two sets of ATAC peaks, both of which showed a classic peak conservation profile displaying higher levels of conservation in sequences close to the centre. Interestingly, we found the differential ATAC regions are less conserved than the non-differential ATACs (Fig. 2E; Table S3). This may relate to greater selective pressure on genomic regions with essential transcription factor binding, and the occurrence of diverse recent mechanisms of gene regulation in differential response.

### Enhancer-derived RNA (eRNA) signatures in different monocyte states

Enhancer-derived RNAs (eRNAs) are a group of RNAs transcribed by RNA polymerase II from transcriptional enhancers, a major type of *cis*-regulatory element in the genome [41] (Fig. S4A). We defined eRNAs based on transcript abundance in distal ATAC peaks [42]. Overall 7.8% of the distal ATAC peaks (771 out of 9,929) have eRNA expression of which 66.9% (516 out of 771) contain CAGE-based eRNAs that were identified by FANTOM5 across different tissue/cell types. We then compared between treatment conditions and found variance in eRNA expression by treatment group on principal component analysis, specifically PC1 explaining 45% of the variance (Fig. S4B). LPS6/6 and LPS24 samples show significant overlap suggesting that they share some similarity in eRNA expression.

**Fig. 3:**
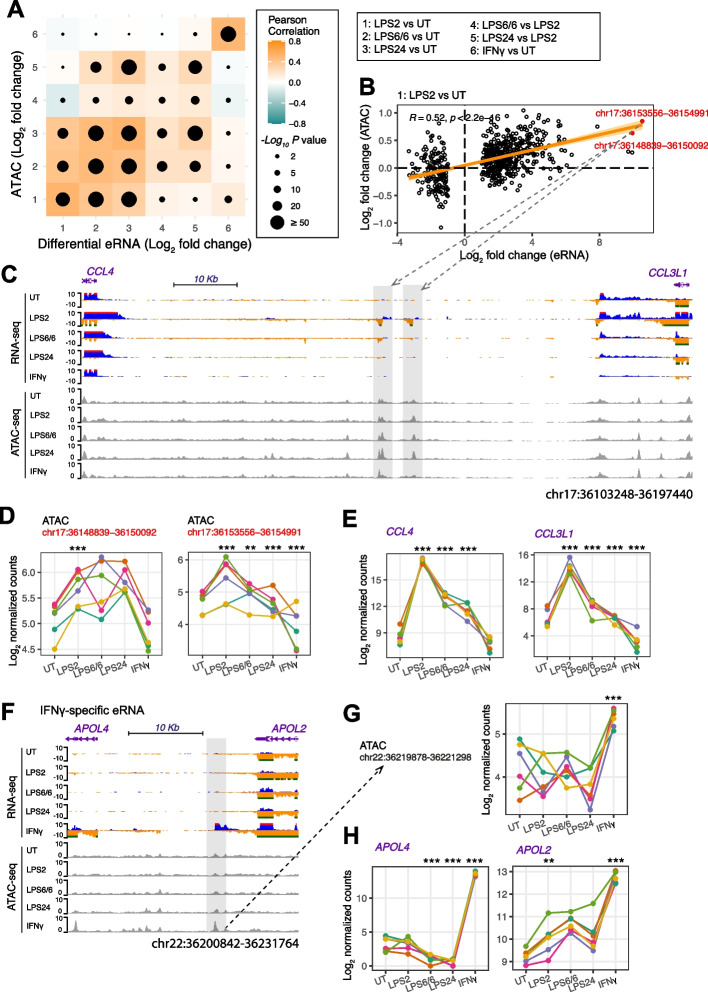
Differential enhancer RNAs and associated chromatin accessibility changes. **A** Correlation of log2 fold change of differential eRNAs and ATAC regions for all treatment types. Pearson’s r and p values are shown. **B** Correlation between log2 fold change of eRNAs and ATAC regions for naive and acute response states, highlighting two genomic loci (chr17:36153556 − 36154991 and chr17:36148839 − 36150092) adjacent to *CCL4* and *CCL3L1* genes with significant chromatin accessibility changes. The orange line represents the direction and strength of the linear relationship between datasets as indicated by the orange line was measured by Pearson's correlation. **C** Genome browser tracks showing differential eRNA expression between LPS2 and LPS6/6 conditions and their chromatin accessibility changes. Average eRNA expression and ATACs for the 6 healthy donors, for each treatment type are shown. Regions with differential eRNAs and ATACs are highlighted in grey. **D** Line plots illustrate chromatin accessibility changes at chr17:36153556 − 36154991 and chr17:36148839 − 36150092 loci for each healthy donor (indicated by different colour dots with linked lines) for different treatment types. *P*-value was calculated by linear regression. *** *p* < 0.001, ** *p* < 0.01. **E** mRNA abundance of *CCL4* and *CCL3L1* genes show an elevated expression of these genes in LPS2 compared to naïve and LPS tolerant states (*** *p* < 0.001). **F** Example of IFNγ-specific eRNAs proximal to differentially expressed genes, *APOL4* and *APOL2*. **G** Line plots illustrate enhanced chromatin accessibility changes at chr22:36219878–36221298 locus and (**H**) transcriptional activation of *APOL4* and *APOL2* genes corresponding to IFNγ treatment. Each donor is indicated by different colour dots with linked lines (*** *p* < 0.001)

We identified differential eRNA specific to different endotoxin treatments **(**Table S4) and a correlation between context-specific eRNA expression and differential ATACs (Fig. 3A and B). This included eRNAs in a gene desert between the chemokine genes, *CCL4* and *CCL3L1* (localising to chr17:36153556 − 36154991 and chr17:36148839 − 36150092) induced most strongly by LPS2, that were present to a reduced extent with LPS6/6 (Fig. 3C); this is correlated with significant chromatin accessibility changes (*** *p* < 0.001; Fig. 3D), and increased *CCL4* and *CCL3L1* mRNA abundance (Fig. 3E). We also observed IFNγ-specific eRNAs proximal to *APOL4* and *APOL2* genes (at chr22:36219878–36221298; Fig. 3F) associated with differential chromatin accessibility (~ 2-fold increase in IFNγ samples compared to naïve group; *** *p* < 0.001, Fig. 3G) and enhanced expression of *APOL4* and *APOL2* genes (compared to the naïve group, ~ 100 and 14-fold increase in IFNγ samples, respectively; *** *p* < 0.001, ** *p* < 0.01; Fig. 3H and Fig. S8A).

### Differential ATAC regions, gene expression and pathway activation by endotoxin state and IFNγ

**Fig. 4:**
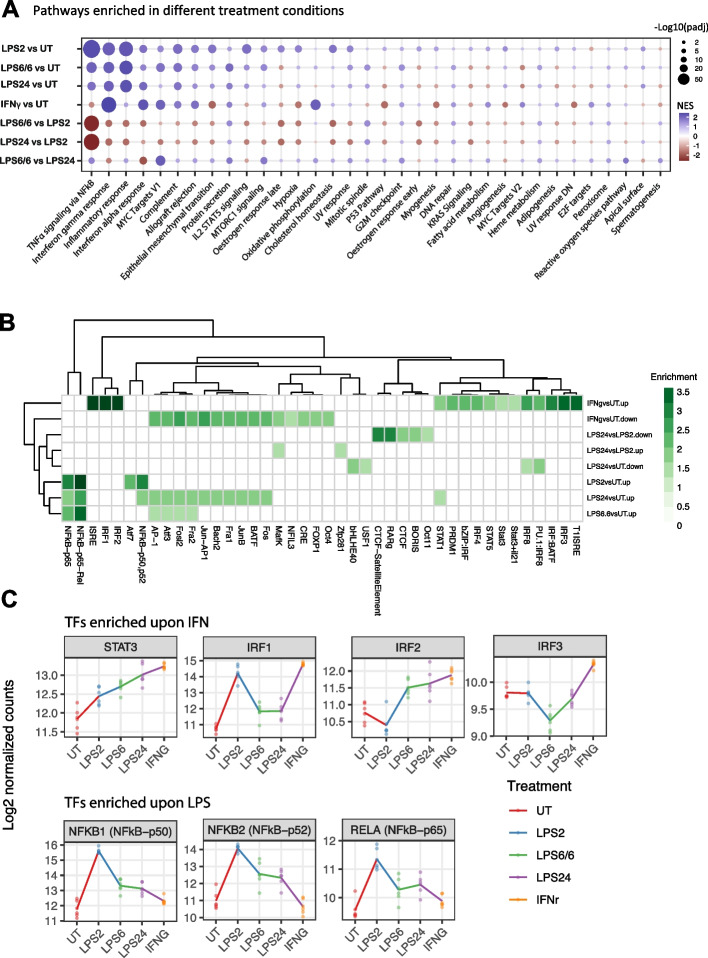
Pathway enrichments between different treatment types and transcription factor activity analysis on integrated ATAC and RNA-Seq datasets. **A** Gene-set enrichment analysis (GSEA) showing the direction and magnitude of expression changes of hallmark gene sets between different treatment contrasts. All expressed genes (*n* = 21,298; genes with counts > 5 in more than 10% healthy donor samples) without an arbitrary DE cutoff are included. Only the top 5 upregulated and top 5 downregulated gene sets for each treatment comparison were included in the analysis. The key genes involved in each pathway and their statistical significance are summarized in Table S5. KEGG pathway database [43–45] used with permission. **B**. Heatmap showing the enrichment of transcription factor binding sites in differential ATAC relative non-differential ATAC regions upon different treatment contrasts. Only the significantly enriched motifs (q < 0.05 and fold change > 1.5) were shown. **C** The expression patterns of the enriched transcription factors across different treatment conditions

We next sought to combine information from the different assay modalities and apply systems biology approaches to understand effects at a pathway and network level. Pathway analysis of our transcriptome profiling across treatment conditions demonstrated differential expression of canonical pathways associated with the acute endotoxin (LPS) and IFNγ responses, most significantly TNFα signalling (by NF-kB and IFNγ response pathways respectively) together with inflammatory response, complement, oxidative phosphorylation (IFNγ), hypoxia (IFNγ), IL2-STAT5 signalling (LPS) and cholesterol homeostasis (LPS), MYC target V1 (IFNγ) (Fig. 4A, Table S5). MYC target V1 and MTORC1 signalling pathways were identified as highly upregulated between LPS6/6 and LPS24, endotoxin tolerant conditions (Table S5).

Endotoxin tolerance (LPS6/6) and chronic exposure to LPS (LPS24) showed relative loss of enrichment for TNF signalling via NFkB (Fig. 4A), reflected in differential expression of constituent genes for this pathway including *IL12B, CSF2, PTX3* and *IL6* (Fig. S5A). Component genes and differentially expressed genes for the TNF pathway are shown for each of the comparator treatment groups (Fig. S5B and C).

Using the differential ATAC regions upon different treatment conditions, we searched the known transcription factor (TF) binding sites in 200 bp regions centred on the differential ATAC peaks (Method), and determined 42 TF motif sequences were enriched (q < 0.05 and fold change > 1.5 relative to non-differential peaks; Fig. 4B), including STAT/IRF/ISRE/T1ISRE regulatory elements that were known to be involved in IFN signalling and NFkB components for LPS pathways, which as expected showed concordant stimulation-specific expression patterns (Fig. 4C).

We observed significant upregulation of genes belonging to the Matrix metalloproteinase (MMP) family during endotoxin tolerance, particularly *MMP1*, *MMP7, MMP9, MMP12, MMP14*, which are involved in the breakdown of extracellular matrix, cell proliferation, adhesion, apoptosis, differentiation and host defence [46]. For example, when compared to naïve and LPS2 states, *MMP1* showed ∼30fold and ∼23-fold increased expression in LPS6/6 condition, respectively (Fig. S6A-C). We also found *CEMIP, SERPINB7, KLHL2*, *ADIPOR1* and *LGALS3* genes were highly induced by LPS6/6 and LPS24 conditions (Fig. S6D-J). Metallothioneins, highly conserved metal-binding proteins that play vital roles in metal ion homeostasis, protection against heavy metal toxicity, modulation of inflammation, DNA damage, cell proliferation and oxidative stress, were highly upregulated in endotoxin tolerance (LPS6/6 and LPS24). In humans, metallothioneins are encoded by a family of genes located on chromosome 16q13 [47], and expression was strongly upregulated in at least 10 genes (Fig. S7) with highest chromatin accessibility changes in *MT-1 M, MT-1F* and *MT-1H* genes (Fig. S7).

**Fig. 5:**
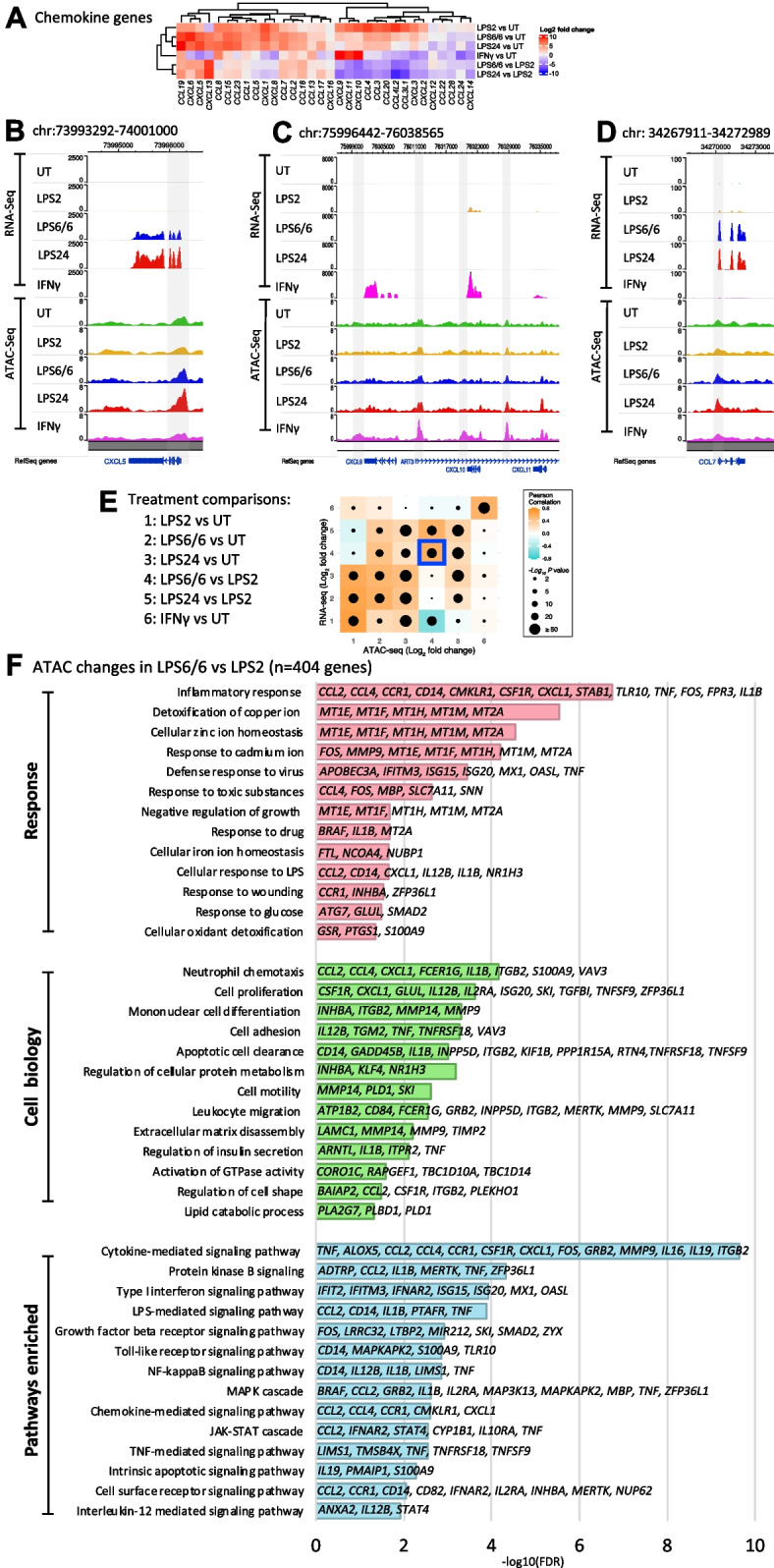
Context-specific chemokine gene expression, and molecular signalling pathways involved in transcriptional and chromatin accessibility changes upon LPS6/6 treatment. **A** Heatmap summarising the differential expression of chemokine genes in different treatment contrasts (also see Table S6). **B**-**D** Genome browser tracks showing chromatin accessibility changes associated (marked in grey boxes) with differential expression of *CXCL* genes in LPS24 and IFNγ treatment conditions, and *CCL7* in endotoxin tolerant conditions. Mean gene expression and chromatin accessibility change for the 6 healthy donors for each treatment condition are shown in RNA-Seq and ATAC-Seq Genome Browser alignments. **E** Correlation between differential ATAC and nearest gene (nGenes) expression to the chromatin peak, highlighting LPS6/6 vs LPS2 condition in a blue box. Pearson’s r and p values are shown. **F** Response type, cellular functions and main regulatory pathways associated with key (FDR < 0.05) genes that had significant chromatin accessibility changes upon LPS6/6 treatment compared to LPS2, acute response

IFNγ induced widespread upregulation of gene expression including *CXCL9, ANKRD22, APOL4, P2RY14, CXCL10, CCL7, UBD, SEPT4, GBP1P1, CXCL11, VCAM1, CALHM6, IDO1, EXOC3L4,* and *HAPLN3* genes (8–13fold increase) compared to the naïve state (Fig. 5A-D and Fig. S8A, C) together with guanylate binding protein genes (*GBP1-6*; Fig. S8B), *MMP25* and *IL32* (Fig. S8D), *ETV7* (Fig. S8E), and classical HLA genes. Differential ATAC peaks on IFNγ treatment were seen for a number of these genes such as *CXCL10* (Fig. 5C)*, GBP5* and *ETV7* together with other loci (Fig. S8B, E; Table S3). Enriched pathways from differential gene expression involved defence response to virus, cellular response to type I IFN, IFN-mediated signalling, and regulation of immune response (Fig. S8F).

Overall, the expression of chemokine genes was highly variable between treatment types, and in some cases associated with concomitant changes in chromatin accessibility (Fig. 5A-D and Table S6).

We proceeded to assess at a genome-wide level the correlation between RNA-Seq and differential ATAC [nearest genes (nGenes) to the chromatin peak] to identify genomic regions with significant chromatin accessibility and gene expression changes involved in response to LPS and IFNγ treatments (Fig. 5E). We found 284, 404, 2,486 genes involved in both significant chromatin accessibility changes and gene expression changes in LPS2 vs UT, LPS6/6 vs LPS2, and LPS24 vs LPS2 contrasts, respectively (Fig. 5F, Figs. S9, 10, Table S7). As expected, this identified pathways associated with inflammatory response, viral defence and cellular response to LPS, and with cytokine and LPS-mediated signalling following acute LPS stimulation (LPS2) (Fig. S9). We specifically focused on the endotoxin tolerance conditions and observed significant enrichment for pathways including inflammatory response, detoxification of zinc, copper and cadmium ions; neutrophil degranulation and chemotaxis, IL6 regulation; and cytokine mediated signalling (Fig. 5F, Fig. S10). Key genes involved in cytokine-cytokine receptor interaction pathways and their differential expression in different treatment conditions are illustrated in Fig. S11-S14.

### Stimulation-specific eQTLs infer causal enhancer-gene relationships

**Fig. 6:**
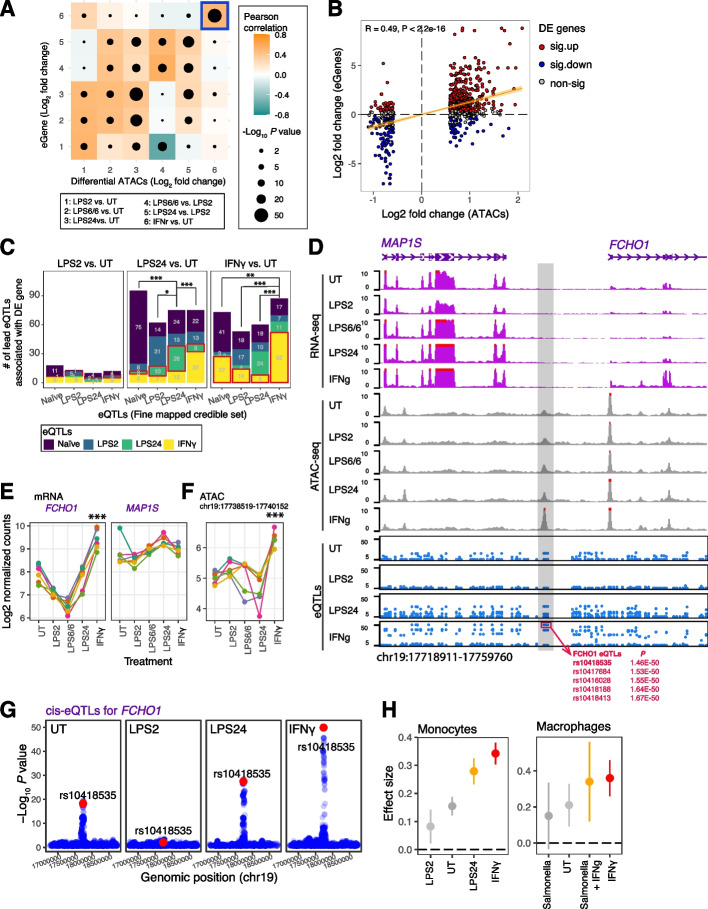
Stimulation-specific eQTLs infer causal enhancer-gene relationships. **A** Correlation of log2 fold change of the differential ATAC peaks and their associated eGenes across the six treatment conditions. **B** Dot plot showing the log2 fold change of ATAC peaks (x axis) and linked eGenes (y axis) upon IFNγ treatment (IFNγ vs UT) in monocytes. Pearson’s r and p value are shown. The significantly differentially expressed genes upon IFNγ (FDR < 0.05) were highlighted in red (upregulation) and blue (downregulation). **C** Bar plots showing the number of fine-mapped lead eQTLs used to link the differential ATACs and differentially expressed eGenes upon each treatment condition. The most significant eQTLs in any monocyte state (*p* < 1e-05; naive, LPS2 and LPS24) were attributed to ATAC peaks, and then restricted to the differential peaks and DE genes identified upon different treatment conditions (left panel: LPS2 vs. UT; middle panel: LPS24 vs. UT; right panel: IFNγ vs. UT), and the number of eQTLs in any monocyte state determined (shown in different colours) that belong to a given fine mapped credible set (x axis). P value was calculated by two-tailed Fisher’s exact test. ***P < 0.001; **P < 0.01; *P < 0.05. **D** *FCHO1* locus showing a differential enhancer upon IFNγ treatment (region highlighted in grey) harbouring IFNγ-specific *FCHO1* eQTLs (red box). **E** mRNA expression of each gene (left panel: *FCHO1*; right panel: *MAP1S*) surrounding the differential enhancer as indicated in (**C**) was compared upon IFNγ stimulation. **F** Enhancer profile was compared upon different treatment conditions with samples from 6 different healthy donors (as colour dots with linked lines). **G** Regional association plots for *FCHO1* eQTLs across different stimulation conditions in monocytes. The SNP rs10418535 is highlighted in red. **H** The effect size of rs10418535 for *FCHO1* in monocytes (left panel) and macrophages (right panel) across the treatment conditions as indicated on the x axis. The points represent the eQTL effect size, and the error bars represent 95% confidence intervals. See also Fig. S15

In order to understand the molecular mechanisms involving individual enhancers in cellular phenotypes and immune signalling, it is important to identify their likely target genes. To do this we intersected evidence of the location of putative regulatory elements from observed ATAC profiles with the location of genetic variants associated with differential expression of specific genes previously mapped in the same stimulation state for human monocytes [28] (as eQTL) to identify candidate target genes (eGenes) for individual potential enhancers. For a given enhancer-eGene pair, we used the eQTL with the most significant association across the treatment conditions. This ensured that the more likely causal eQTL and its associated gene was selected within each ATAC peak, only one gene was assigned to each peak, and the context-specificity of the eQTL association was also introduced in the analysis. When we linked those differential enhancers to genes through eQTLs, we found the coincident relationships between the enhancer activity and eGene expression in each individual treatment condition (Fig. 6A). For example, amongst the 2,066 ATAC peaks that were differential upon IFNγ stimulation, 862 peaks have at least one cis-eQTL identified in monocytes at naïve or stimulated states (LPS2, LPS24 or IFNγ treatments) [28]. These peaks were assigned to a total of 689 unique eGenes whose expression was positively correlated with the ATAC abundance (Fig. 6A highlighted in blue box and Fig. 6B). Overall, this analysis revealed 1,946 unique differential ATAC associated with 1,340 eGenes through 1,937 eQTLs (Table S8).

We next determined the probability that the eQTLs from the context-specific datasets were causal for the ATAC-eGene associations using gene expression profiles and the fine-mapping eQTL sets [48], and found that the lead eQTLs identified in a given context were more like to reside within ATACs linking differentially expressed genes identified in the same treatment condition relative to lead eQTLs in other contexts (Fig. 6C). For example, among differential ATAC colocalising with monocyte eQTL for any state, we determined 59.8% (52 out of 87) of the lead eQTLs upon IFNγ resided in differential ATACs and differentially expressed genes that were identified in IFNγ versus UT condition, compared to only 13.3% (8 out of 60) of the lead eQTLs upon LPS24 (P = 9.3e-09, OR = 9.5; Fig. 6C right panel). These likely causal interactions include the gene *FCHO1* that has clustered lead eQTLs (rs10418535 in complete linkage disequilibrium with rs10417684 rs10416028, rs10418188 and rs10418413 in Europeans) located in an IFNγ-specific enhancer region (Fig. 6D). The heightened enhancer abundance upon IFNγ was associated with clearly IFNγ-induced expression of *FCHO1* (Fig. 6F and E left panel), but not the other surrounding gene *MAP1S* (Fig. 6E right panel). Similarly, the lead eQTL rs58295246 resided in a more accessible ATAC peak (Fig. S15A-B) and was associated with heightened expression of *FAM20A* upon LPS24 (Fig. S15C). Interestingly, the eQTL rs10418535 was also reported to be a chromatin accessibility quantitative trait locus (caQTL) residing an ATAC peak linking *FCHO1* in naïve macrophages [49]. The C allele of the SNP was associated with attenuated chromatin accessibility, reduced *FCHO1* expression and increased risk of developing a complex inflammatory disease, coronary artery disease. Reassuringly, the lead variant rs10418535 for *FCHO1* expression showed the strongest association in monocytes treated with IFNg compared with naïve or LPS-treated cells (Fig. 6G and H left panel), and showed a high degree of concordance in macrophages (Fig. 6H right panel).

In order to provide a broader overview of monocyte eQTLs and independent overlapping-GWAS loci we intersected eQTLs in ATAC regions with current GWAS lead SNPs reported in the NHGRI-EBI GWAS catalog (*P* < 5e-08; https://www.ebi.ac.uk/gwas/). The eQTLs within differential ATAC peaks of different monocyte states and overlap with GWAS SNPs are listed in Table S9.

## Discussion

In this paper we have mapped the response to endotoxin and IFNγ at the level of gene expression and chromatin accessibility, with a particular focus on two models of endotoxin tolerance, which has provided a detailed atlas for these disease relevant immune states. We have shown how there is widespread differential exon usage, and enhancer-derived RNA signatures specific to different monocyte states that correlated with chromatin accessibility changes. These data have allowed context-specific definition of putative regulatory elements, which, where differential to activation state, are predominantly distal to genes and less conserved. Moreover, we have shown how these data can be integrated with genetic mapping of expression quantitative traits to identify likely enhancer modulated genes and further interpret GWAS.

Endotoxin tolerance is considered as a protective mechanism of the host against systemic inflammation, however the resulted immunosuppressive state is associated with high risks for secondary infections which leads increased mortality. The endotoxin response in human primary monocytes to innate immune activation provides an important model system to identify individual variation in response and inform progress towards a precision medicine approach, that would aim to maximize the effectiveness of therapy for a given person or population group depending on their response state [34, 50]. Monocytes have been the subject of intensive transcriptional and enhancer profiling [51, 52]. This includes application of CAP analysis of gene expression and histone modifications enabling the promoter-enhancer landscape to be defined in specific monocyte subpopulations [53] and in monocyte-derived macrophages linking identified inducible enhancers with susceptibility to inflammatory bowel disease from GWAS [54] and more broadly how co-expression can be used to fine map causal variants [17]. The involvement of epigenetic mechanisms in higher-order chromatin interactions that modulate gene transcription, contributing to molecular and cellular phenotypes disease relevance is highlighted by studies showing reversal of LPS-induced tolerance in human monocyte-derived macrophages at the level of distal element histone modification and transcriptional reactivation by β-glucan [36]. Further examples are studies showing DNA methylome [55] remodelling of patients with sepsis caused by gram-negative bacteria operates through JAK2-STAT pathway coupled with IFNγR upon autocrine/paracrine IFNγ release [56]; persisting monocyte changes in pneumonia patients involving lipid metabolism through an integrated transcriptomic and DNA methylation analysis [57]; and that LPS-treated human monocytes show reduced levels of histone H3K27ac and H3K4me1 at promoters and enhancers of phagocytic and lipid metabolism genes [36]. These regulatory elements are typically tissue- and context-specific, regulating gene expression in different cellular states and treatment conditions. However, the coordination between gene expression changes and its regulatory epigenetic landscape for chromatin accessibility, and their precise balance required to maintain immune homeostasis, is an area of active research in the field with specific contexts such as following an endotoxin challenge and specific disease states currently incompletely understood.

Our analysis of chromatin accessibility and gene expression in monocytes highlighted both epigenetic and transcriptomic relatedness and differences between different immune cells and subsets [42, 53, 58]. Monocytes with repeated exposure to endotoxin were characterised by enhanced chromatin accessibility (80% of the differential ATAC peaks were upregulated) with differential expression of genes mainly involved in responding to detoxification and environmental stress-associated cell damage. Elevated expression of metallothionein genes involved in both physiological and xenobiotic heavy metal detoxification was identified as a key biomarker of this immunosuppressive state. Metallothionein expression levels are highest in the liver and kidney, which act as primary organs in eliminating toxic substances [59]. This raises the possibility that the body may use similar molecular signalling and physiological pathways involved in heavy metal detoxification, to achieve immune homeostasis following an endotoxin challenge.

Our findings that the majority of differential ATACs (64%) were located in distal regions (> 3 Kb away from annotated TSSs) vs proximal locations (36%) are consistent with other studies reporting for example 25% of all ATAC-seq peaks were located in promoter regions [60]. These data are consistent with enhancers being located distantly in a linear genome but spatially (in 3D) proximal to their targeted genes. These cis-regulatory elements are known to have significantly higher conservation than randomly selected genomic sequences [61, 62]. Relative to promoters, other cis-regulatory regions including enhancers are less conserved and evolve rapidly [62, 63]. Our data reveal that the stimulation-specific ATAC regions are more likely to be distal enhancers and might have faster functional evolution than non-differential ATACs.

eRNAs transcribed from active enhancers have been shown to associate enhancer-promoter interactions, and initiate the waves of transcription factor binding and mRNA expression during cellular differentiation or activation [58, 64–66]. Several potential action mechanisms underlying the eRNA activity on enhancer-gene interactions have been proposed, including its roles in stabilising the chromatin looping [67], promoting elongation [68], regulating histone acetylation [69] and chromatin-remodeling events *in trans* [70]. The recently proposed model for contact-independent enhancer-promoter communication [71] suggests that these eRNAs may have additional functional roles that do not influence the spatial chromatin loops with target promoters. For example, a local concentration gradient of diffusible eRNAs arising from an active enhancer may promote the transcription of its nearby gene by modulating transcription factor acetylation, resulting in the closest promoter being more likely to be activated, which is consistent with studies showing linking the regulatory variants to genes through proximity achieved the highest precision and recall than other predictions such as chromatin looping data [72]. These findings highlight how eRNA levels can be used to quantify the enhancer activity and inform the high-confident enhancer-gene interactions. For example we found IFNγ-induced eRNAs that are regulated by an open chromatin region, chr22:36219878–36221298, also involved in enhanced expression of Apolipoprotein L2 (*APOL2*) and Apolipoprotein L4 (*APOL4*) genes that act in lipid exchange and transport throughout the body, as well as in reversing cholesterol transport from peripheral cells to the liver [73].

Recent eQTL studies have identified genetic variants associated with the majority of human genes in diverse tissue types upon the steady-state, stimulated and disease settings [28, 74, 75], and statistical fine-mapping [76] has provided credible sets of putative causal regulatory variants and has been broadly used to enhance our understanding of molecular mechanisms underlying context-specific gene expression and to GWAS. However, multiple likely causal regulatory eQTLs may be in high linkage disequilibrium and reside within a large non-coding genomic region, and one variant often exhibits strong associations with multiple candidate eGenes. Using both gene expression and chromatin accessibility data, we identified that hundreds of putative causal context-specific eQTLs were located in differential ATAC regions in monocytes for multiple immune stimulated conditions and showed coincident relationships between eQTLs, ATAC abundance and gene expression. For example, we highlighted the IFNγ-specific eQTL rs10418535 associated with coronary artery disease. The risk allele of SNP was associated with reduced expression of *FCHO1* and predicted to attenuate chromatin accessibility via disrupting the binding of PU1/IRF [49]. Our results suggest that re-activation of *FCHO1* or its associated enhancer may be a potential therapeutic target for coronary artery disease.

Toll-like Receptor (TLR) signalling plays a vital role in the response to infection and restoring immune homeostasis. Apart from encoding canonical mRNAs that produce proteins to promote inflammation, many genes in the TLR signalling pathway also encode alternative mRNA isoforms that produce proteins that have been shown to act as negative inhibitors of TLR signalling, providing a mechanism for terminating persistent TLR signalling and initiating endotoxin tolerance during inflammation [77]. Supporting this, we found that LPS tolerant states induce more alternative splicing events (differential exon usage) compared to LPS acute or IFNg stimulation states.

Limitations of this study include the lack of power to analyse inter-individual differences in response and genetic variation directly in epigenetically profiled healthy donors; the absence of other multi-omic assay types that would be informative for epigenetic state notably histone modifications and specific transcription factors using chromatin immunoprecipitation as well as high resolution chromosomal conformation capture mapping; the lack of resolution of specific monocyte subtypes; understanding of more granular dose and time-specific differences in the nature and kinetics of the epigenetics of the tolerance response; characterisation of ethnic and population differences in observed epigenetic profiles by donor; and the need for functional validation of specific enhancer elements using for example CRISPR interference [42].

Our data demonstrate the importance of chromatin accessibility and enhancer activity in determining the transcriptional response to differing innate immune stimuli in human monocytes and how they may associate with the high degree of context-specificity observed for induced eQTLs. Integration of our findings with other multiomics data such as DNA methylation, histone modification and chromatin interactions will unravel the nature and extent of the full functional genomic landscape which drives the innate immune response during endotoxin tolerance.

## Materials and methods

### Sample collection, monocyte separation and preparation of cells for stimulation assays

A total of 6 healthy donors (3 males and 3 females with a median age of 30 years) of British Caucasian origin were recruited from the Oxfordshire area after written informed consent. PBMCs were purified from 90-100 ml of whole blood from each donor using Ficoll-Paque density gradient method. Cells were washed three times in Hanks’ balanced salt solution without Ca^2+^ and Mg^2+^ (Invitrogen) and the total PBMCs count was determined using the haemocytometer. CD14^+^ monocytes were isolated from PBMCs by positive selection with Magnetic-activated cell sorting MicroBeads (Miltenyi Biotec) which is designed to provide a sample of ~ 99% purity. Monocytes were resuspended in T25 flask containing prewarmed RPMI-1640 medium (Sigma) supplemented with 20% Foetal Calf Serum (FCS; Sigma), penicillin/streptomycin (Sigma), and L-glutamine (Sigma) at a density of 1 × 10^6^/ml. Cells were left on a resting state overnight (16 h) at 37 °C, 5% CO2.

Following the resting period, cells were transferred into a 50 ml falcon tube, centrifuged at 250 g for 10 min at room temperature and resuspended in 2.5 ml of prewarmed RPMI-1640 medium, supplemented with 20% FCS, penicillin/streptomycin, and L-glutamine. Then the cell count was taken to determine the viable cell fraction left following resting and that was equally divided into 5, 5-ml nonadherent polypropylene cell culture tubes (BD Biosciences). The cell density in each tube was adjusted to 1 × 10^6^/ml using prewarmed RPMI-1640 supplemented with 20% FCS, penicillin/streptomycin, and L-glutamine prior to begin the stimulation assays.

### Context-specific immune stimulations

CD14^+^ monocytes from each individual was subjected to five stimulation conditions; Naïve untreated (monocytes kept in the incubator for 24 h without any treatment), LPS2 and LPS24 (monocytes exposed to ultrapure LPS (20 ng/ml; Invivogen) for either 2 h or 24 h, respectively), LPS6/6 (monocytes that were first exposed to a low dose of LPS (2 ng/ml) for 6 h followed by washing with prewarmed culture media and re-exposing the cells to a higher dose of LPS (20 ng/ml) for another 6 h), and IFNγ (monocytes exposed to IFNγ (catalog# 285-IF, R&D Systems) at a concentration of 20 ng/ml for 24 h.

Experiments were terminated simultaneously, ensuring identical incubation periods for all samples. Cell samples were resuspended in 1 ml of PBS/1% FCS and a final cell count was taken for each stimulation condition to determine the viable cell number left following stimulations. All experiments were completed within 48 h of blood sample collection. Finally, cell aliquots from each stimulation condition were preserved for ATAC-Seq and RNA-Seq analyses. An aliquot of 1–2 × 10^6^ cells was stored in RLT plus reagent (Qiagen) for RNA extraction (*n* = 30). For ATAC-Seq, two replicates for each stimulation condition from each donor was performed (*n* = 60) and a fresh sample of 50,000 cells were immediately processed for Omni-ATAC-Seq.

### RNA extraction and sequencing

Total RNA from cells stored in the RLT reagent was extracted using the *AllPrep DNA*/*RNA*/*miRNA Universal Kit (Qiagen) according to manufacturer’s instructions. To* minimize contamination with genomic DNA, an additional DNase I (Qiagen) digestion step was performed. Quantity and quality checks of extracted RNA was performed using High Sensitivity Qubit system (Life Technologies) and RNA Screen Tape Assay on Agilent 4200 *TapeStation* System, respectively. RNA-seq library was prepared using a standardised rRNA depletion and dUTP protocol. cDNA was synthesized using *SuperScript III First*-*Strand Synthesis System* for RT-PCR (catalog#: 18080–051). Adapter ligated and amplified cDNA libraries were then multiplexed and sequenced on HiSeq4000 platform (Illumina) at the Oxford Genomics Centre (Wellcome Trust Centre for Human Genetics, Oxford, UK).

### ATAC-Seq

Omni-ATAC-seq was performed as previously described [78, 79]. Cells (50,000) were spun down at 500* g* at 4 ^0^C for 5 min, washed with 50 μL of cold 1 × PBS buffer, and lysed in 50 μl of cold lysis buffer (10 mM Tris–HCL pH 7.4, 10 mM NaCl, 3 mM MgCl_2_, 0.01% Digitonin, 0.1% Tween-20 and 0.1% NP40) for 3 min. Nuclei were washed with 1 ml Wash buffer (10 mM Tris–HCL pH 7.4, 10 mM NaCl, 3 mM MgCl_2_ and 0.1% Tween-20) and spun down at 500 g for 10 min at 4 °C. The cell pellet was resuspended in the Transposition Mixture (25 μl 2 × TD buffer, 2.5 μl Tagment DNA Enzyme I; Nextera DNA Sample Prep Kit; Illumina, 16.5 μl PBS, 0.5 μl 1% Digitonin, 0.5 μl 10% Tween-20, 5 μl nuclease-free H_2_O) and incubated for 30 min at 37 °C,1000 RPM. The reaction was stopped by adding 250 μl of DNA Binding Buffer (Qiagen MinElute Kit). DNA was purified using MinElute PCR cleanup kit (Qiagen) and the DNA was eluted into 23 μL of Elution Buffer. To determine the appropriate cycle number for library amplification, qPCR was carried out using 2 μL of purified DNA with 1 μL each Nextera primer (Ad1_noMX/Ad2.1; 25 μM), 10 μL 2X NEB Next PCR Master Mix, 0.2 μL 100X SYBR Green, and 5.8 μL nuclease-free H_2_O. The libraries were dual-indexed using the optimised primers for ATAC-seq [78, 79]. The fragments were amplified using 2 × NEB Next PCR master mix and 1.25 M of custom Nextera PCR primers. The libraries were purified using a MinElute PCR cleanup kit (Qiagen), quantified using Qubit assay (ThermoFisher), quality-controlled using Agilent 4200 *TapeStation* System and subjected to high-throughput sequencing on the Illumina HiSeq 4000 Sequencer at the Oxford Genomics Centre (Wellcome Centre for Human Genetics, Oxford, UK).

### Genome-wide epigenetic & transcriptomic profiling of samples

#### RNA-Seq data analysis

We quantified transcription for both gene expression and exon usage. We trimmed the sequencing adapters form the fastq files using Trim Galore (version 0.6.2), and then mapped the reads to the hg38 reference genome (Homo_sapiens.GRCh38.dna.primary_assembly.fa file, downloaded from Ensembl; release 84) using the HISAT2 (version 2.1.0). The aligned Binary-sequence Alignment Format (BAM) files were used to determine the gene counts via featureCounts (version 1.6.2) and GENCODE annotations (release 31). For gene differential expression analysis, the raw read counts were used as input into the R package DESeq2 (version 1.28.1) for pair-wise comparisons. We filtered out genes that have less than 5 reads mapped in more than 90% of the samples, retaining 21,298 genes for downstream analysis. Genes with fold change > 2 and FDR < 0.05 as per condition were considered as differentially expressed. For exon usage analysis, we first generated an exon annotation gtf file using Ensemble transcriptome annotations (release 84) and dexseq_prepare_annotation2.py script from (https://github.com/vivekbhr/Subread_to_DEXSeq) and R package DEXSeq (version 1.32.0) [80]. We then used the aligned RNA-seq BAM files to count reads aligned each exon using featureCounts with flags ‘-p -f -O -s 2 -t exonic_part’. The exonic regions with read counts ≥ 10 in at least 10% of samples were used as input into DEXSeq for pair-wise comparisons. Exon usage with fold change > 2 and FDR < 0.05 as per condition were considered as differentially expressed. The bigwig files normalized by RPKM (Reads Per Kilobase per Million mapped) for visualization in WashU Epigenome Browser were generated using the bamCoverage function of deepTools (version 3.3.1) [81].

Principal Component Analysis was done using Python package sklearn and all plots were also done in Python using the packages Matplotlib, Seaborn, Venn and NetworkX. Gene interaction networks were found using xSubneterGenes from the R package XGR [82].

#### ATAC-seq analysis and the association between ATAC profiles and gene expression

Reads from ATAC sequencing were aligned to human genome assembly hg38 using bowtie2 (version 2.2.5), and the resulting BAM files were filtered to remove non-uniquely mapped reads, non-properly paired reads, reads mapped to mitochondrial chromosome, duplicate reads and reads with a mapping quality score < 30 using Picard (version 2.0.1) and Samtools (version 1.9). ATAC peaks were called using MACS2 (version 2.1.0) with flags ‘–nomodel –shift -100 –extsize 200’. The normalized bigwig files showing the average sequencing depth across donors were generated using wiggletools and wigToBigWig. For differential chromatin accessibility analysis, we filtered out the peaks that overlay the ENCODE Blacklist (hg38 liftover version of the one downloaded form http://hgdownload.cse.ucsc.edu/goldenpath/hg19/encodeDCC/wgEncodeMapability/) and with peak call p value > 1e-05. Peaks called in ≥ 30% of samples were defined as recurrent, and merged as a list of coordinates to count the overlapping reads in each treatment condition using htseq-count (version 0.6.1). We used the raw counts and DESeq2 to determine the differential ATACs upon each condition (fold change > 1.5, FDR < 0.05). We examined the potential batch effect by principal component analysis, and called the differential ATACs adjusting the baseline difference across the donors in DESeq2 formula (design =  ~ Treatment + Donor). For the heatmaps of ATAC signal, we first generated the normalised bigwig files using bamCoverage function of deepTools and the size factor of each sample computed by DESeq2 based on all recurrent peaks across conditions. We then plotted the results using the computeMatrix and plotHeatmap functions of deepTools.

To link the ATAC peak to their nearest genes (nGenes), we first generated the gtf annotation file for the monocyte expressed genes (maximum TPM ≥ 0.5 across samples; *n* = 21,919) based on GENCODE annotations (release 31), and then used the HOMER [83] (version 4.10) findMotifsGenome.pl command with default parameters to map each peaks to their closest gene transcription start sites. To attribute the peaks to eQTL genes (eGenes), we localised the eQTLs (P value < 1e-05) within the peak regions, and selected the most significant eQTL-gene pair across all the treatment conditions. The eQTL summary data and fine-mapped credible sets for CD14 monocytes with or without immune stimulations [28] were downloaded from eQTL Catalogue (https://www.ebi.ac.uk/eqtl/) [48]. Enrichment of TF biding motifs within the differential ATAC peaks was calculated using the HOMER [83] (version 4.11) findMotifsGenome.pl command with default parameters.

#### eRNA analysis and visualisation

eRNA analysis was performed as described in [42]. Briefly, we took the midpoint of each ATAC recurrent peak, and extended with 1 Kb from left and right. We filtered out those regions that overlap with the gene boundaries (± 3000 bp from both transcription start site and end site) of the 21,919 monocyte expressed genes (maximum TPM ≥ 0.5 across samples). We used these distal ATAC regions (*n* = 9,929) as coordinates to count the number of uniquely-mapped total RNA-seq reads in each condition via the multicov function of bedtools with default setting (version 2.27.0). We used the raw counts and DESeq2 to quantify and compare the eRNA expression across the treatment conditions. eRNAs with fold change > 2 and FDR < 0.05 as per condition were considered as differentially expressed. To visualise the eRNA profile in WashU Epigenome Browser, we first extracted strandedness of the RNA-seq reads using Samtools with flags ‘-f 128 -F 16’ and ‘-f 80’ for the forward reads, and ‘-f 64 -F 16’ and ‘-f 144’ for the reverse reads, and then generated the normalized bidirectional bedgrapgh files using Bedtools and Bedops. The proportion of distal ATAC peaks with eRNA expression was based on maximum CPM >  = 1 across 30 samples with different treatment conditions). We downloaded the updated set of CAGE based human eRNAs defined by FANTOM5 (https://fantom.gsc.riken.jp/5/datafiles/latest/extra/Enhancers/), and converted the coordinates to hg38 version using *liftOver* and restricted to the distal ones (> 3 Kb from the gene boundaries), and determined the proportion containing CAGE-based eRNAs identified by FANTOM5 across different tissue/cell types.

#### Pathways analysis and KEGG pathway colour mapping

GSEA (Gene-set enrichment analysis) was performed using the R package fgsea (v1.14.0). Gene sets were retrieved from Molecular Signatures Database (MSigDB v7.4) using msigdbr (v7.4.1). Genes were ranked by sign(log2 fold change) x –log10(P value) upon each treatment condition. The top 10 enriched pathways in ranked gene lists (top5 with up-regulated genes and top5 with down-regulated genes) were used for visualization. Colour mapping of gene expression (by fold-change) was performed using KEGG mapper colour pathway [43–45, 84] and XGR software 82.

#### Evolutionary conservation

We measured the evolutional constraint of the sequence by using the phyloP score [85], the -log(p value) under a null hypothesis of neutral evolution: negative scores indicate fast-evolving while positive scores indicate conservation. We downloaded the bigwig file containing the PhyloP conservation score derived from the alignment of 100 vertebrate genomes per base from the UCSC browser (http://hgdownload.cse.ucsc.edu/goldenpath/hg38/phyloP100way/hg38.phyloP100way.bw). To calculate the conservation scores for each ATAC peaks, we first took the midpoint of each recurrent ATAC peak, expanded it using a ± 1 Kb window and divided the 2 Kb coordinates into 10 bp bins. We then determined the average conservation scores for each bins across the peaks using the PhyloP score bigwig file and UCSC BigwigAverageOverBed tool (version2).

### Supplementary Information

**Additional file 1: Table S1.** Differentially expressed genes in different treatment conditions. 

**Additional file 2: Table S2.** Differential differential exon usage in different treatment conditions. 

**Additional file 3: Table S3.** Differential ATAC regions in different treatment conditions.

**Additional file 4: Table S4.** Differential eRNA in different treatment conditions.

**Additional file 5: Table S5.** Top pathways enriched for Hallmark gene sets differentially expressed between treatment conditions.

**Additional file 6: Table S6.** Expression levels of chemokines genes in LPS2, LPS6/6 and LPS24 conditions.

**Additional file 7: Table S7.** Pathway enrichment for genes showing significant chromatin accessibility changes and gene expression changes in different treatment conditions.

**Additional file 8: Table S8.** eGenes, eQTL and differential ATAC by treatment condition.

**Additional file 9: Table S9.** ATACs with monocyte eQTLs and GWAS SNPs.

**Additional file 10: Figure S1.** Differentially expressed genes in LPS treatment groups. **Figure S2.**  Comparison of differential gene expression signatures from proinflammatory and anti-inflammatory macrophage lineages and LPS tolerance. **Figure S3.** Differential exon usage among treatment conditions. **Figure S4.** Differential enhancer RNA (eRNA) expression. **Figure S5.** Differentially expressed genes among treatment types. **Figure S6.** Key genes upregulated during endotoxin tolerance. **Figure S7.** Gene expression and chromatin accessibility changes of Metallothionein genes in different treatment conditions. **Figure S8.** Genes induced by IFNg treatment. **Figure S9.** Top pathways involved in transcriptional and chromatin accessibility changes in LPS2 vs UT. **Figure S10.** Top pathways involved in transcriptional and chromatin accessibility changes in endotoxin tolerant conditions. **Figure S11.** Key cytokine families involved in cytokine-cytokine receptor interactions pathway. **Figure S12.** Class I helical cytokines involved in cytokine-cytokine receptor interactions pathway. **Figure S13.** Class II helical cytokines and interleukin genes involved in cytokine-cytokine receptor interactions pathway. **Figure S14.** TNF and TGF- β family genes involved in cytokine-cytokine receptor interactions pathway. **Figure S15.** Stimulation-specific eQTLs infer causal enhancer-gene relationships. 

## Data Availability

ATAC-seq and RNA-seq raw fastq files are available at the European Genome-Phenome Archive (EGA) with accession number EGAS00001007362 (access is managed by a Data Access Committee). The processed data including the raw and normalised counts, the bedGraph and bigWig files for genome-wide signal data are available at Zenodo (https://zenodo.org/record/8158923).
